# Absolute Electrical Impedance Tomography (aEIT) Guided Ventilation Therapy in Critical Care Patients: Simulations and Future Trends

**DOI:** 10.1109/TITB.2009.2036010

**Published:** 2009-11-10

**Authors:** Mouloud A. Denaï, Mahdi Mahfouf, Suzani Mohamad-Samuri, George Panoutsos, Brian H. Brown, Gary H. Mills

**Affiliations:** 1 Department of Automatic Control and Systems EngineeringUniversity of Sheffield Sheffield S3 7GG U.K.; 2 Department of Medical PhysicsUniversity of Sheffield Sheffield S10 2JF U.K.; 3 Department of Critical Care and AnaesthesiaNorthern General Hospital Sheffield S5 7AU U.K.; 4 University of Sheffield SheffieldS3 7GG U.K.

**Keywords:** Biomedical imaging, blood gas, electrical impedance tomography (EIT), mechanical ventilation, respiratory system

## Abstract

Thoracic electrical impedance tomography (EIT) is a noninvasive, radiation-free monitoring technique whose aim is to reconstruct a cross-sectional image of the internal spatial distribution of conductivity from electrical measurements made by injecting small alternating currents via an electrode array placed on the surface of the thorax. The purpose of this paper is to discuss the fundamentals of EIT and demonstrate the principles of mechanical ventilation, lung recruitment, and EIT imaging on a comprehensive physiological model, which combines a model of respiratory mechanics, a model of the human lung absolute resistivity as a function of air content, and a 2-D finite-element mesh of the thorax to simulate EIT image reconstruction during mechanical ventilation. The overall model gives a good understanding of respiratory physiology and EIT monitoring techniques in mechanically ventilated patients. The model proposed here was able to reproduce consistent images of ventilation distribution in simulated acutely injured and collapsed lung conditions. A new advisory system architecture integrating a previously developed data-driven physiological model for continuous and noninvasive predictions of blood gas parameters with the regional lung function data/information generated from absolute EIT (aEIT) is proposed for monitoring and ventilator therapy management of critical care patients.

## I. Introduction

Mechanical ventilation (MV) is an essential component in supportive therapy of critical care patients and patients with respiratory disorders. MV aims to improve pulmonary gas exchange via an adequate tidal volume ($V_{T}$) delivered at a suitable ventilatory/respiratory rate (RR). Oxygenation is improved by either raising the fraction of inspired oxygen ($\hbox{FiO}_{2}$) or increasing the positive end-expiratory pressure (PEEP) that helps to prevent recruited lung units collapsing at end-expiration. Another strategy, which aids the opening of potentially collapsing airways, is to prolong the inspiratory time by increasing the ventilator's inspiration-to-expiration (I:R) ratio. $\hbox{CO}_{2}$ elimination is improved by increasing the minute volume either via an appropriate setting of $V_{T}$ or RR.

Although MV can be a lifesaving intervention for many intensive care (ICU) patients, it has been associated with potential complications known as ventilator-induced lung injury (VILI) [1], [2]. The choice of appropriate ventilator mode and settings can improve the benefit-to-risk ratio of MV by providing adequate gas exchange while reducing the risk of VILI [3], [4]. However, known bedside measures to guide the clinician in adjusting MV settings are limited in that they tend to give global information regarding the performance of the lungs. Arterial blood gas analysis and airway pressure–volume (PV) graphical waveforms have been the gold standard clinical practices for assessing the acid–base balance, lung function, and guiding the titration of MV in critically ill patients. These are combined with measurements derived from pressure, flow, and volume, which give information about the mechanical properties of the lungs and chest wall. However, these methods are only able to provide an indication on the overall lung function, and thus fail to provide full information about the regional lung behavior. To date, chest imaging has relied on bedside X-ray radiography and the gold standard computed tomography (CT), which provides comprehensive images of the morphologic structures of the lungs and shows ventilation distribution with high spatial resolution. However, during these procedures, the patient is exposed to a substantial dose of radiation, and in the case of CT, the patient needs to be transported to the radiology department, which is a high risk process in the unstable critically ill. Indeed, the risks may be so high that the investigation is not carried out at all. Even if possible, the practical considerations mean that CT is an occasional investigation usually only repeated every few days at most. It is costly and both time and labor intensive. The process will require waiting for the scanning time to be available and will usually take up an hour or two of medical, nursing, and technician time (potentially depleting staff resources on the ICU itself), as well as radiology staff time, portering involvement, and occupying transfer equipment and ventilators. The last point is particularly important, as changing from the ICU ventilator to a transport ventilator will result in a brief period of loss of PEEP, with the potential for lung derecruitment or more subtle changes in ventilator pressures or flows, which may be deleterious for the patient. Absolute electrical impedance tomography (aEIT) provides a cheap, potentially continuous form of monitoring the behavior of the lungs, which may reveal changes and trends in regional ventilation. For example, progressive loss of ventilation in one lung may lead to the early detection of an endotracheal tube that has slipped down either the right or left main bronchus, leading to obstruction and underventilation of the opposite lung, or may suggest the development of some other pathology in the deteriorating lung, such as a developing pleural effusion compressing the lung or worsening atelectasis due to a disease process such as a pneumonia. Long-term electrode application can be irritant for the skin (although not more than ECG monitoring, which is almost invariably in place). There are practical problems with maintaining good electrical contact and coping with the wires. However, practice and timely application when patients are being rolled as part of routine patient care can go a long way to reduce these problems.

EIT is a promising noninvasive monitoring tool that allows real-time imaging of regional ventilation of the lungs at the bedside. The first clinical EIT images were obtained from the Sheffield Group [5] who used a simple backprojection algorithm to reconstruct cross-section images of the thorax. The equipment used 16 electrodes and produced an image resolution of 104 pixels. A filtered backprojection method, similar to the one used in CT imaging, was later implemented by the same group in order to improve the spatial resolution of the reconstructed images.

Among the clinical applications of EIT being investigated by other groups are the monitoring of internal bleeding, the measurement of gastric emptying, the measurement of cardiac output, and imaging of the brain [6]. However, the monitoring of the pulmonary function is arguably the most promising application of EIT [7], [8]. Many current ongoing research studies are being directed at demonstrating the ability of EIT to image regional lung ventilation in a clinical setting [9]–[11]. A software package EIDORS/GREIT (electrical impedance tomography and diffusion based optical tomography reconstruction software/Graz consensus reconstruction algorithm for electrical impedance tomography) implementing different methods for the solution of the forward and inverse problems in EIT using finite-elements modeling techniques is available for public use [12].

The purpose of this paper is to demonstrate the potential usage and ability of EIT to assess regional ventilation distribution in the lungs using a comprehensive physiological model. This combines a model of respiratory mechanics, a model of lung absolute resistivity as a function of air content, and a 2-D finite-element model (FEM) of the thorax with 16 electrodes to simulate EIT current injection and voltage measurements. The resulting physiological model can simulate different scenarios of acute respiratory distress syndrome (ARDS) lungs and reproduce consistent images of lung ventilation distribution in response to different PEEP levels. Finally, a new advisory system using multisource data fusion architecture is proposed for monitoring and ventilator therapy management of critical care patients.

The remainder of the paper is organized as follows. Section II covers the basics of EIT. Section III focuses on the description of the physiological model and its principal components. Sections IV and V present simulation studies with different scenarios of ARDS lungs. Finally, Section IV presents the prospective advisory system prototype that is currently being developed by the authors.

## II. Principles of EIT and Image Reconstruction Techniques

**Fig. 1.**
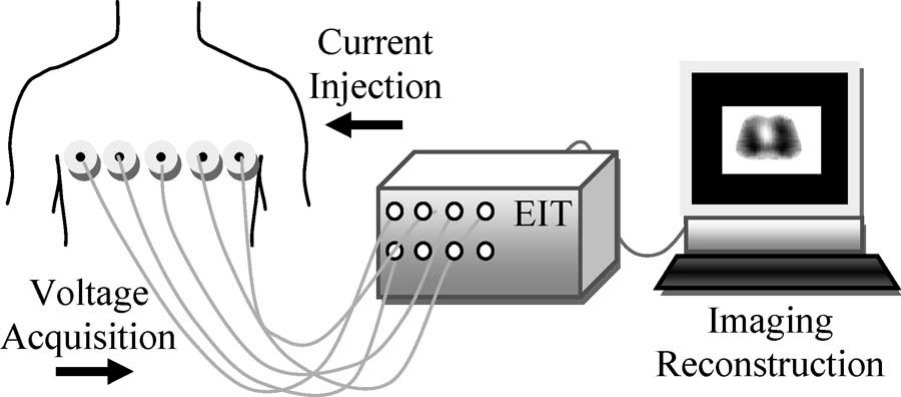
Typical EIT system with 16 electrodes for current injection and voltage acquisition.

In EIT, current patterns are injected into the body via surface electrodes, and boundary voltages are measured to reconstruct a cross-sectional image of internal distribution of the conductivity or resistivity. A typical EIT system that uses a set of electrodes attached to the surface of the chest at about 4–5 cm above the xyphoid process is depicted in Fig. 1.

Most EIT equipments use alternating currents with amplitude and frequency varying from various amperes and low frequency for geophysical application to 1–10 mA and 1 kHz–1 MHz for medical applications.

There are many ways to apply current and measure the resulting voltages. The most popular data collection strategy is the so-called adjacent or four electrode, where current is applied to an adjacent pair of electrodes and the resulting voltages between the remaining 13 pairs of electrodes are measured.

**Fig. 2.**
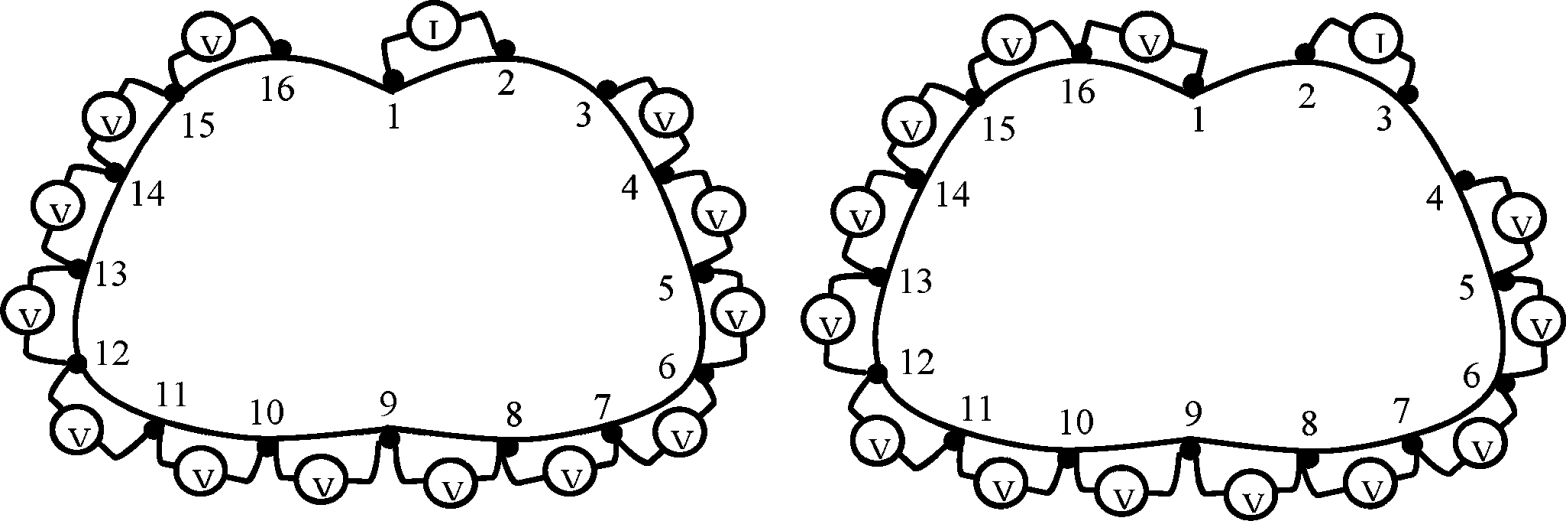
Adjacent measurement configuration with 16 equally spaced electrodes.

In a 16-electrode system, current is injected through electrode pair (1,2) and the resulting boundary voltages differences are measured from the electrode pairs (3,4), (4,5), …, (14,15), (15,16), as shown in Fig. 2.

This procedure is repeated 16 times with current injected between successive pairs of adjacent electrodes until all 16 possible pairs of adjacent electrodes have been used to apply the known current. This is called a frame of data and will produce 16 × 13 = 208 measurements.

Mathematically, the problem of recovering the conductivity or resistivity within a body $\Omega$ from the surface measurements of currents and potentials is a nonlinear inverse problem and severely ill-conditioned. The following approximation (Laplace’ equation) is often used in EIT as long as the frequency is in the range of 0–10 kHz in which biological tissue exhibits distinct conductivity values [5]. However, many researchers are investigating potential solutions to the full Maxwell's equations [13]

$$\nabla \cdot (\sigma \nabla u) = 0. \eqno{\hbox{(1)}}$$

The Neumann boundary conditions on $\partial \Omega$, the boundary of $\Omega$ are formed by fixing the normal current $J_{\vec n}$ at every point on $\partial \Omega$

$$\sigma {{\partial u} \over {\partial \vec n}} = \cases{ {J_{\vec n},} \hfill&$ {\hbox{under}\,\hbox{the}\,\hbox{electrodes}}$\hfill\cr {0,} \hfill&$ {\hbox{elsewhere}}$\hfill }
\eqno{\hbox{(2)}} $$

where $\sigma$ is the conductivity, $u$ is the potential, $\vec J$ is the density of the injected current, and $\vec n$ is the normal vector to the surface. For the uniqueness of the solution to this problem, a variety of assumptions has to be made [14].

These equations can be solved either with analytical methods or via numerical techniques using finite-element or finite-difference techniques [15], [16]. A systematic approach for solving the reconstruction problem is to solve the *forward problem*, which consists of finding a unique effect (voltages) resulting from a given cause (currents) via a mathematical or physical model (conductivity distribution). The process of recovering the conductivity distribution within the body from the applied currents and measured boundary potentials is known as the *inverse problem* in EIT. There are two approaches for solving the image reconstruction problem in EIT. *Static* reconstruction produces an image of the absolute conductivity distribution of the medium based on one set of measurements. *Dynamic* or *difference* imaging attempts to recover the change in resistivity based on measurements made at two different time periods.

The quality of the reconstructed images depends on: 1) the number of electrodes and data collection strategy and 2) the reconstruction algorithm employed. The most popular data collection strategy is the so-called adjacent or four electrode described previously. The type of reconstruction algorithm ranges from the simplest linearized single-step method to a computationally intensive iterative technique.

## III. Physiological Model of Ventilated Lungs and EIT

**Fig. 3.**
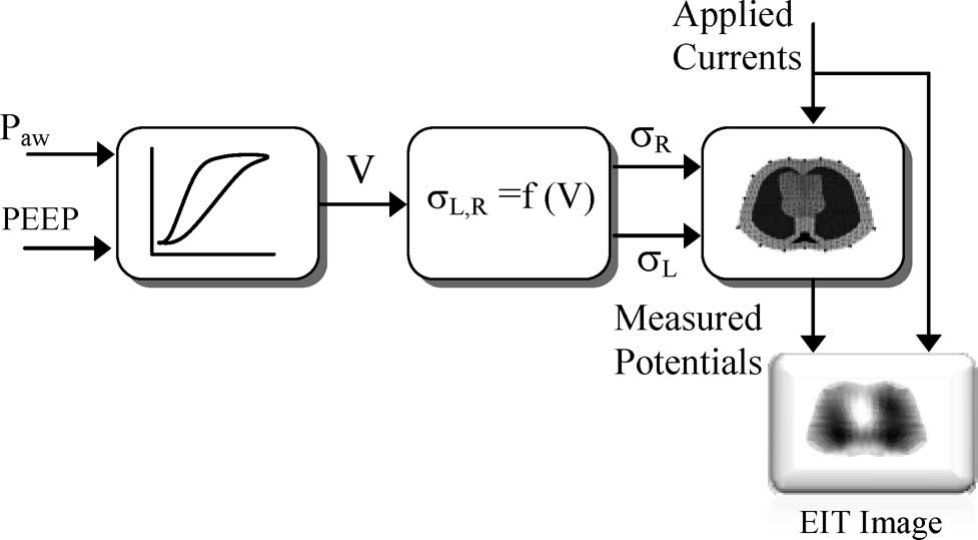
Structure and components of the physiological model. PEEP = positive end-expiratory pressure, $P_{\rm aw}$ = airway pressure, $V$ = lung volume, and $\sigma _{L}$, $\sigma _{R}$ = left and right lung relative conductivities.

The model structure shown in Fig. 3 includes a model of the respiratory mechanics, a physiological model describing the relationship between the assumed lung volume ($V$) and the left ($\sigma _{L}$) and right ($\sigma _{R}$) lung conductivities, and a finite-element cross-section model of the thorax on which the EIT current injection and voltage measurements are performed.

A detailed description of these model components is presented in the following sections, but suffice to say here that the input(s) and output(s) mappings of Fig. 3 should not be too sensitive to noise and other uncertainties provided that special care is exercised when eliciting the relevant models that should have generalizing properties. In this way, the low-frequency properties of such models may act as low-pass “filters” against the earlier disturbances/uncertainties.

### A. Model of the Lung Mechanics

A simple physiological model of the lung mechanics has been proposed by Hickling [17], [18]. The model is based on the hypothesis that lung inflation/deflation is predominantly caused by recruitment/derecruitment of the lung alveoli. The lung is modeled as multiple units or alveoli, which are distributed into compartments characterized by different superimposed pressure (gravitational pressure due to lung weight). In the upright position, the gravitational pressure increases linearly from the uppermost (independent region) compartment to the lowermost (dependant region) compartment. In the supine position, the superimposed pressure increases from the ventral compartment (independent region) to the dorsal compartment (dependant region).

The lung units are described by their compliance curve, which gives a nonlinear relationship between the applied pressure and the lung unit volume. The following equation is used to model this relationship [19]:

$$V = V_0 \left({1 - e^{- P{\rm log}\left({2/h} \right)} } \right) \eqno{\hbox{(3)}}$$

where $V$ is the lung volume, $V_{0}$ is the maximum volume assumed during tidal breathing, $P$ is the pressure, and $h$ is the half-inflation pressure.

**Fig. 4.**
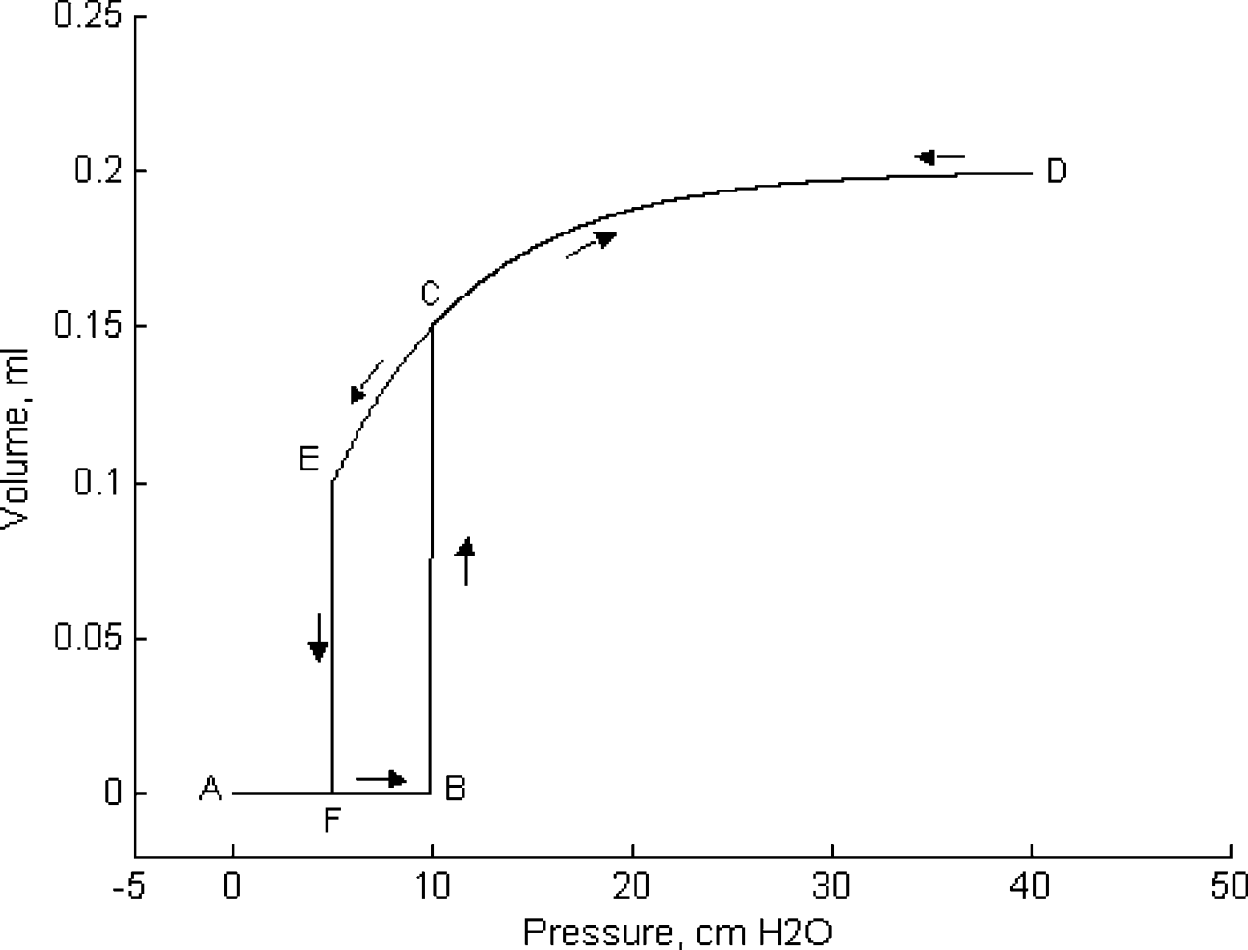
Mechanics of inflation (path ABCD) and deflation (path DEFA) of a single lung unit. TOP and TCP coincide with points B and F, respectively.

In the model, the lung unit can assume only two possible states: recruited (or open) and derecruited (or closed) [17], [20]. These two states are governed only by the threshold opening pressure (TOP), which is the critical pressure above which the lung unit pops open, and the threshold closing pressure (TCP) below which the unit collapses. The model uses normally distributed TOP and TCP pressures with a specific mean and standard deviation (SD), which may be adjusted to reflect the heterogeneous characteristic of alveoli under different abnormal lung conditions such as ARDS [20], [21]. Fig. 4 illustrates the mechanics of a single alveolus during inflation and deflation. During inflation (inspiration), when the applied pressure exceeds the TOP, the lung unit pops open and assumes a volume according to (3). During deflation (expiration), the lung unit collapses and its volume becomes zero when the applied pressure falls below the TCP.

The lung volume at a given pressure can then be calculated by adding up the contributions of the recruited lung units in the different compartments at that specific pressure. The model parameters used throughout are listed in Table I [17].

**Table I:** Baseline Parameters of the Lung Mechanics Model [17]

Parameters	Value
Number of alveoli per compartment	9000
Number of compartments	30
Gravitational pressure (cmH_2_0)	0 to 14.5
Vo (litres)	0.5
h (cnH_2_0)	4.9

### B. Absolute Resistivity Lung Volume Relationship

Nopp et al. [22] developed a detailed model for human lung and used it to determine lung tissue resistivity as a function of frequency. Their model used a cube-shaped alveolus and included components for blood, cellular membrane, endothelial and epithelial cells, and extracellular and intracellular fluids. The overall density of lung tissue is

$$\rho _{{\rm lung}} = {{W_{{\rm lung}} } \over {V_{{\rm air}} + V_{{\rm tissue}}}} \eqno{\hbox{(4)}}$$

where $W_{\rm lung}$ is the lung weight, and $V_{\rm air}$ and $V_{\rm tissue}$ represent the volumes of air and tissue, respectively. The ratio ${{V_{{\rm air}} } \mathord{\left/ {\vphantom {{V_{{\rm air}} } {V_{{\rm tissue}}}}} \right. \kern-\nulldelimiterspace} {V_{{\rm tissue}}}}$ is defined as the filling factor (FF). Substituting this in (4) gives

$$\rho _{{\rm lung}} = {{\rho _{{\rm tissue}} } \over {FF + 1}} \eqno{\hbox{(5)}}$$

where $\rho _{{\rm lung}}$ denotes the density of the lungs condensed matter that has been fixed to 1050 kg⋅m^−3^ in the model [23].

If $W_{\rm lung}$ is known, then the lung density can be calculated as follows:

$$\rho _{{\rm lung}} = {{\rho _{{\rm tissue}} } \over {\left({{{V_{{\rm air}} } \mathord{\left/ {\vphantom {{V_{{\rm air}} } {W_{{\rm lung}}}}} \right. \kern-\nulldelimiterspace} {W_{{\rm lung}}}}} \right)\rho _{{\rm tissue}} + 1}} \eqno{\hbox{(6)}}$$

The lung density $\rho _{{\rm lung}}$ as a function of absolute lung resistivity (AbR) has been obtained by Nopp et al. [22] as follows:

$$\rho _{{\rm lung}} = 3.12 - 3.24 \times [\ln ({\rm AbR})]^{0.3} + 0.81 \times [\ln ({\rm AbR})]^{0.6} \eqno{\hbox{(7)}}$$

Hence,

$${\rm AbR} = 1.74 + 194.3 \times e^{- 24.69\rho _{{\rm lung}} } + 40.04 \eqno{\hbox{(8)}}$$

The left and right lung conductivities are then obtained as $\sigma _L = {\rm AbR}^{- 1} W_{{\rm LL}}$ and $\sigma _R = {\rm AbR}^{- 1} W_{{\rm RL}}$, with $W_{{\rm LL}}$ and $W_{{\rm RL}}$ being the respective weights of the left and right lung. These values have been set to $W_{{\rm LL}} = 633$ g and $W_{{\rm RL}} = 583$ g [24].

### C. Model of EIT

**Fig. 5.**
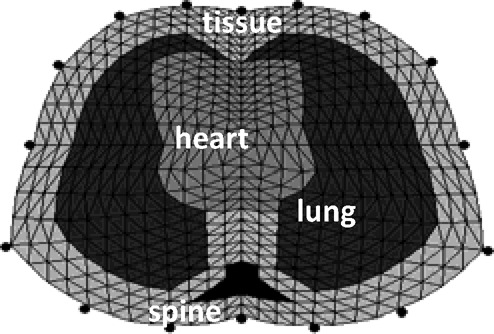
Thorax finite-element-based model with 576 elements, 313 nodes, and 16 electrodes (adapted from [12]).

To solve the inverse problem, one needs to solve the forward problem for some assumed conductivity distribution and compare the generated voltages with those obtained from real measurements. The finite-element method was employed for the numerical solution of (1) and (2) by subdividing the 2-D cross section of the thorax into a finite number of triangular elements. The electric potential is expressed as a linear combination of the nodal basis functions. Each element is assumed to be homogenous and having the same conductivity. The FEM shown in Fig. 5 was used to simulate the subject's cross section of the thorax.

The FEM was divided into four regions of different conductivities that were fixed to their basal values, except those of the left and right lungs that were varied according to Nopp's model.

It is worth noting that using 2-D models in EIT assumes that the currents injected are confined to a 2-D electrode plane. However, since the EIT problem is inherently 3-D, the potential profiles resulting from these 2-D assumptions are expected to be significantly different from those obtained in a 3-D domain. There are extra computational efforts and complexities involved when dealing with 3-D models; however, these can be overcome with the currently available hardware and computational power.

## IV. EIT-Based Monitoring of Ventilated ARDS Patients

In ARDS, the lungs become stiffer and present a heterogeneous distribution of the lung units even within a same lung region [25]. Moreover, clinical studies using CT scans have revealed that the lung units' threshold pressures (TOP and TCP) obey a normal (Gaussian) distribution [21]. In the model, mean and SD of TOP and TCP pressures were given the values shown in Table II to simulate different intensities of ARDS [26]. The mean indicates the pressure at which the maximum of recruitment (TOP) and derecruitment (TCP) of lung units occurs, whereas SD describes the spread of the lung units' population with respect to the TOP and TCP. Moreover, the functional residual capacity (FRC), which is the amount of air that stays in the lungs at the end of a normal expiration during tidal breathing, is also reduced in ARDS-affected lungs. Estimated values for FRC under the degrees of ARDS conditions considered are given in Table II.

**Table II:** Threshold Opening (TOP) and Closing (TCP) Pressures for the Simulated ARDS Scenarios [26]

Degree of ARDS	TOP (cmH20)		TCP (cmH20)		FRC (litres)
	Mean	SD	Mean	SD	
Normal (no ARDS)	4.5	2	2	2	2.4
mild	10	2.9	2.5	2.4	2.2
moderate	14.5	3.8	4.5	2.9	1.8
severe	24.5	4.8	13	3.8	1.5

**Fig. 6.**
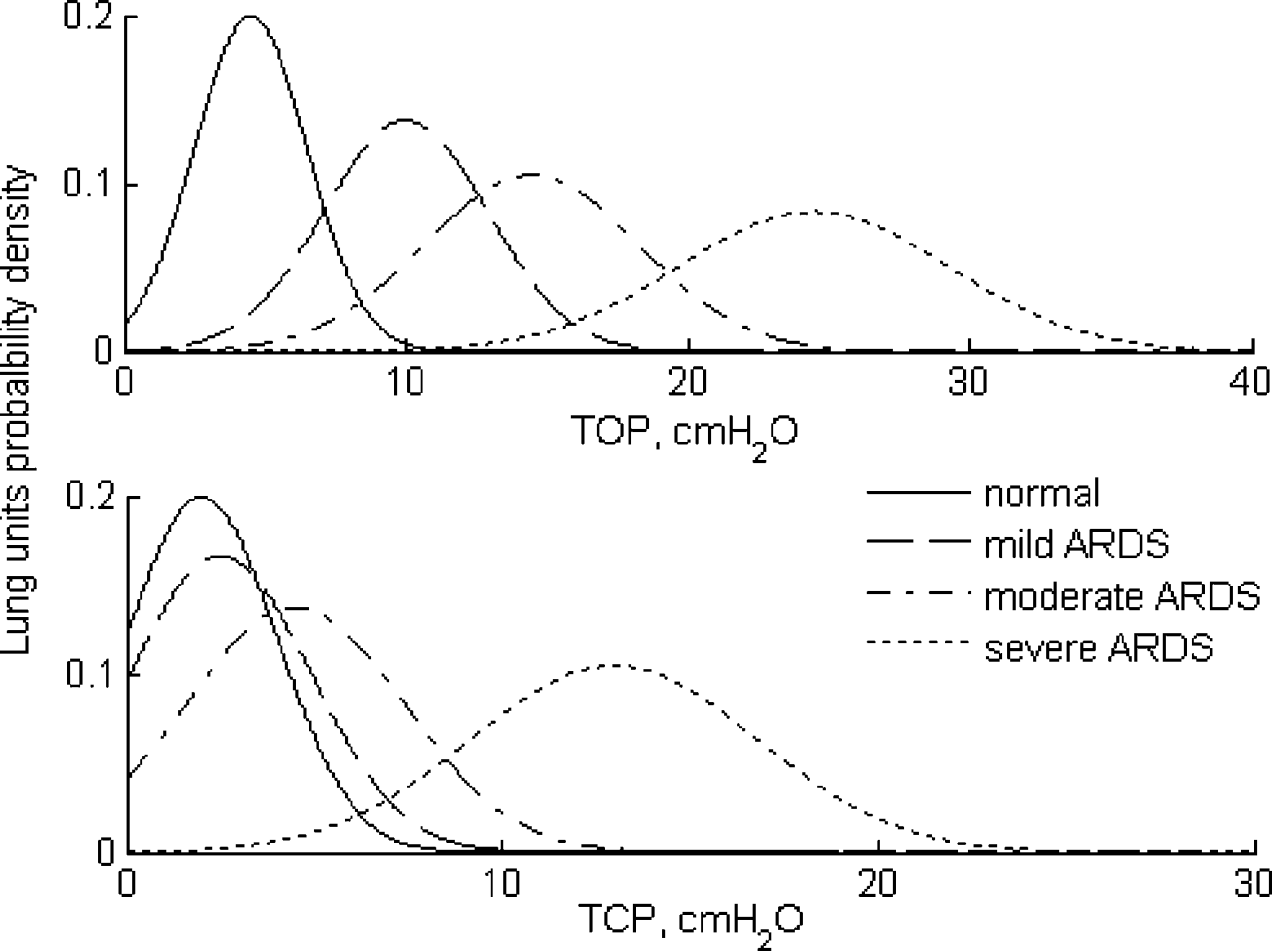
Gaussian distribution of threshold opening (TOP) and closing (TCP) pressures of normal and ARDS conditions (mild, moderate, and severe).

Since ARDS-affected lungs require a higher pressure to inflate to the same volume compared to normal lungs, the mean values of the threshold pressures tend to shift toward higher pressures as the intensity of ARDS increases. In terms of lung mechanics, this shift results in a reduction in lung compliance. The SD, on the other hand, reflects the recruitment rate and lung compliance. Fig. 6 shows the plots of the Gaussian distributions of TOP and TCP pressures for the classes of simulated lung conditions given in Table II.

A tidal breathing cycle is simulated by traversing up (inflation) and then down (deflation) the airway pressure Paw range in small steps from PEEP to peak inspiratory pressure (PIP), and then, from PIP to PEEP, respectively.

**Fig. 7.**
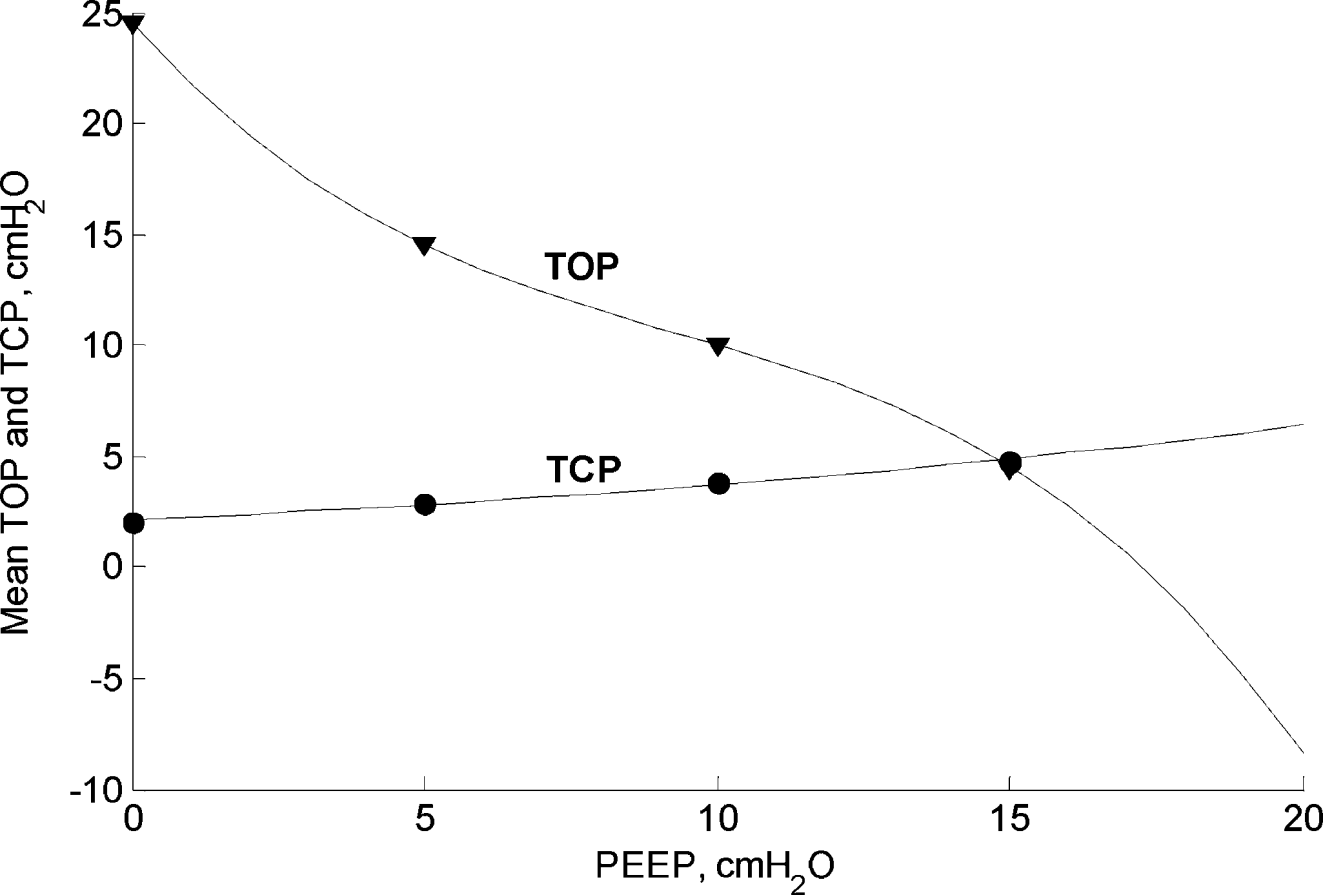
TOP and TCP mean shift against PEEP.

PEEP is expected to produce an upward shift in the *PV* curves, which physiologically represent the volume contribution from the recruited lung units. In the model, this has been simulated by shifting the mean values of the TOP and TCP distributions toward lower and higher pressure ranges, respectively [20]. To reproduce this feature, PEEP values (0, 5, 10, 15 $\hbox{cmH}_{2}\hbox{O}$) were fitted to the means of TOP and TCP for the simulated ARDS lung categories defined in Table II. The resulting equations are plotted in Fig. 7.

**Fig. 8.**
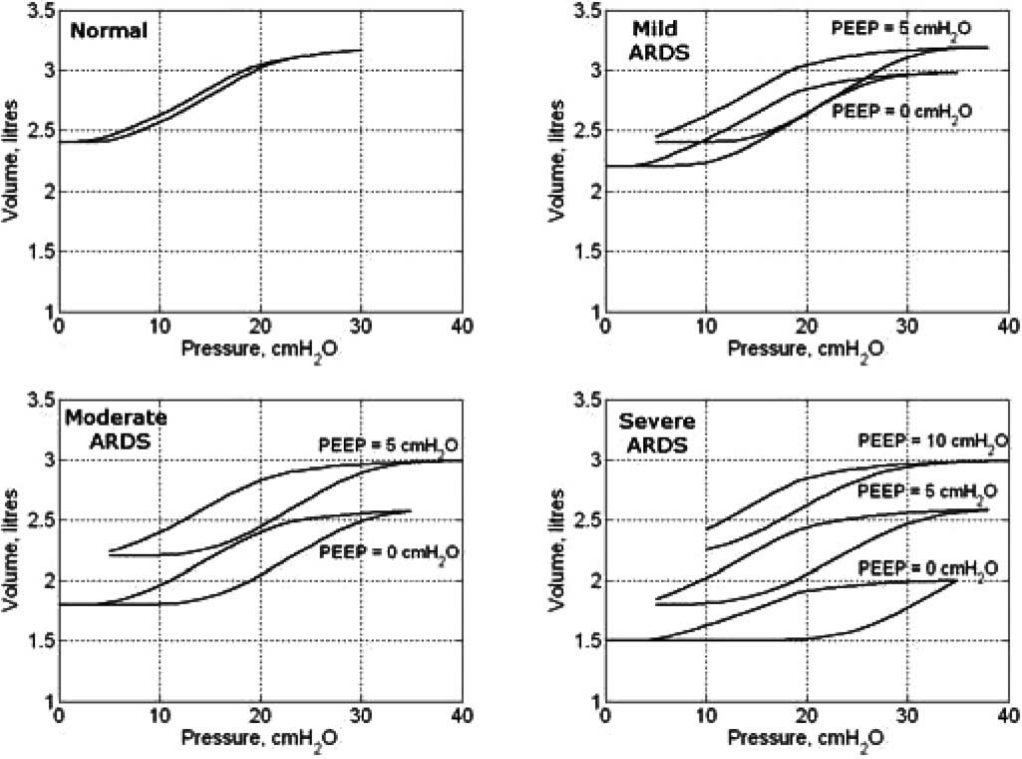
*PV* curves for the simulated normal (healthy) and ARDS conditions (mild, moderate, and severe).

Fig. 8 shows the *PV* curves obtained for simulated healthy and ARDS-affected lungs. In ARDS, the *PV* curves are right-shifted and characterized by larger hysteresis between the inflation and deflation limbs, which is reflected by the difference in TOP and TCP pressures.

**Fig. 9.**
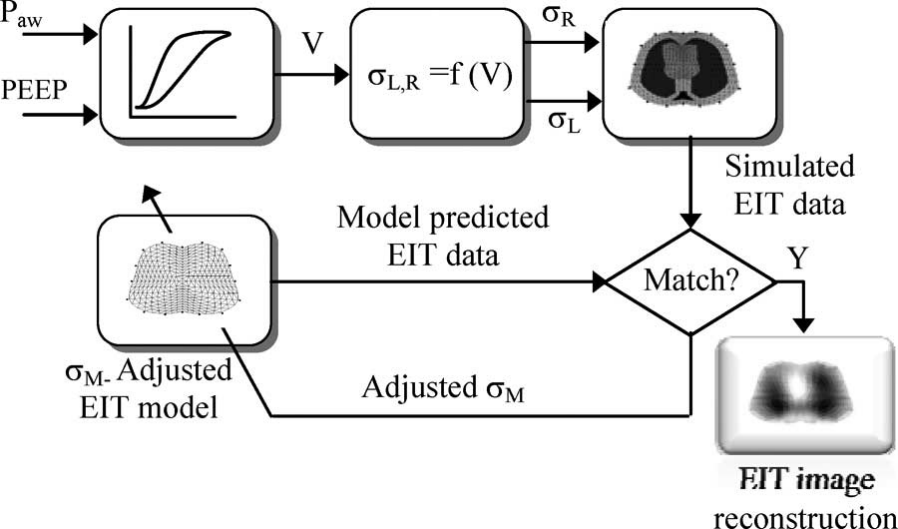
Simulation setup of ventilated ARDS lungs and EIT image reconstruction.

The physiological model of the lung mechanics simulating different degrees of ARDS conditions is incorporated with the EIT model in the simulation setup of Fig. 9.

EIT numerical solution involves updating the conductivity distribution until the predicted and measured voltages match each other within a desired accuracy. The Gauss–Newton iterative algorithm implemented in [12] was also used in this simulation study. The model is cycled through the assigned airway pressure, and at each pressure step, the calculated lung volume is used to set the new absolute conductivities of the left and right lungs on the thorax model of Fig. 5. EIT data are then generated using adjacent drive patterns with an injected current of 5 mA. These measured EIT data are compared to those predicted from another FEM using the same drive and measurement patterns until some desired accuracy has been achieved. On convergence, the reconstructed image of the lung conductivity distribution is displayed. The image sequence is obtained by calculating conductivity changes from a reference state, which defines the basal conductivities for the lungs and the surrounding organs shown in Fig. 5.

**Fig. 10.**
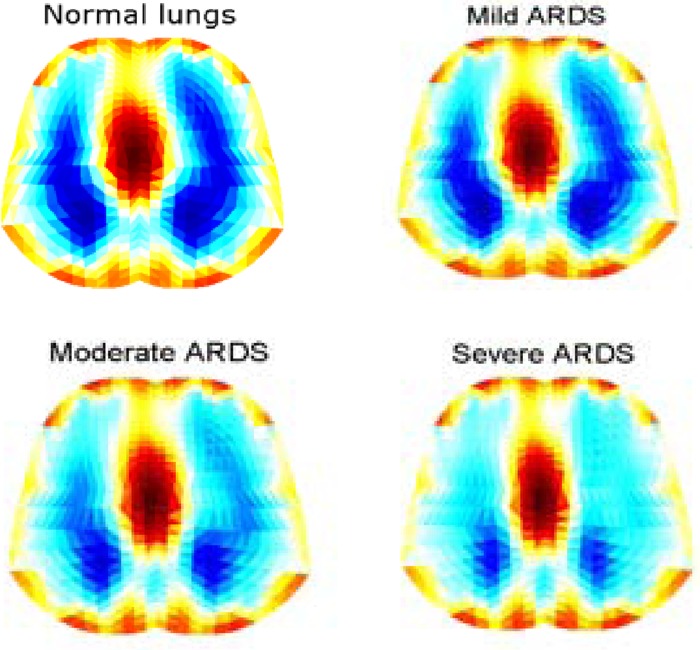
Reconstructed EIT images at end inspiration for normal and ARDS (mild, moderate, and severe) lungs.

Fig. 10 shows the reconstructed EIT images at end-inspiration during tidal breathing for the simulated normal and ARDS lung models. Conductivity values were scaled between 0.2 (nonconductive) and 1.0 (conductive) to produce this image contrast between aerated (dark blue) and nonaerated lung regions (red).

**Fig. 11.**
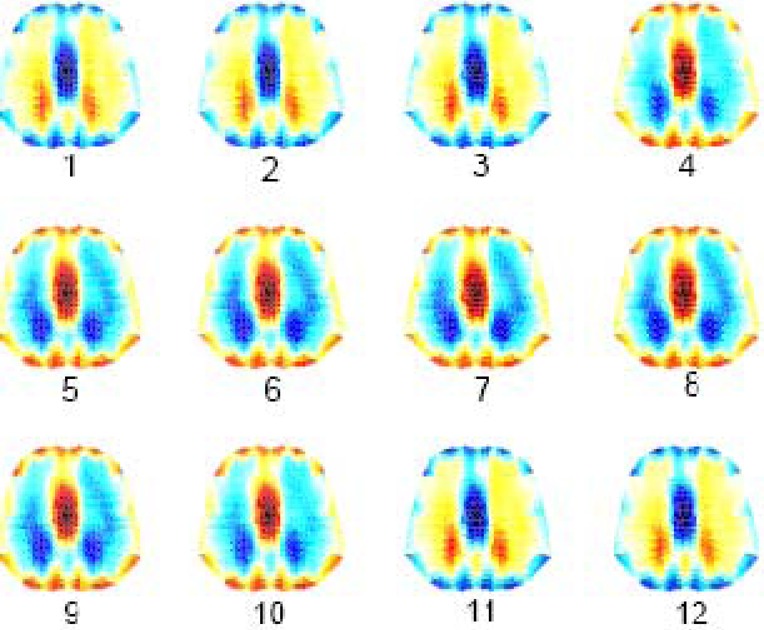
EIT image slices of a breath (expiration-inspiration-expiration) related to moderate ARDS model. PEEP = 0 $\hbox{cmH}_{2}{\kern-1pt}\hbox{O}$ and PIP = 40 $\hbox{cmH}_{2}{\kern-1pt}\hbox{O}$.

Fig. 11 shows the sequence of lung image slices (progressing from left to right and top to bottom) reconstructed from a simulated breath (expiration–inspiration–expiration) related to the moderate ARDS model with the ventilator PEEP and PIP set to 0 and 40 $\hbox{cmH$_{2}$O}$, respectively. Red lung regions correspond to expiration at FRC and dark blue lung regions correspond to inspiration. This image contrast is the result of the simulated changes in the conductivity of the left and right lungs. Lung inflation has begun at slice number 4, confirming the results obtained with the static *PV* curves of Fig. 8.

**Fig. 12.**
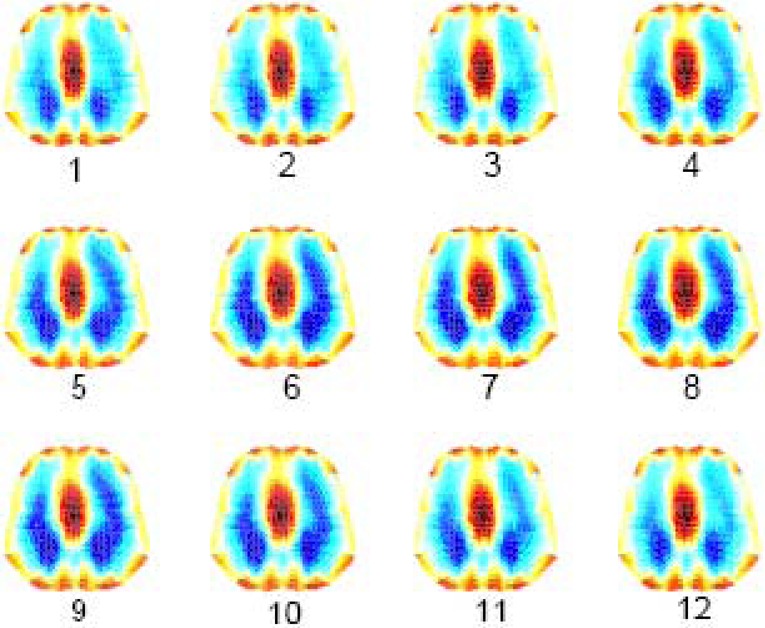
EIT image slices of a breath (expiration–inspiration–expiration) related to moderate ARDS model. PEEP = 5 $\hbox{cmH}_{2}{\kern-1pt}\hbox{O}$ and PIP = 40 $\hbox{cmH}_{2}{\kern-1pt}\hbox{O}$.

In Fig. 12, PEEP was set to 5 $\hbox{cmH$_{2}$O}$ with all the other parameters maintained to their previous values. Those lung units recruited during lung inflation and having TCP pressures below the PEEP value selected remain open at end-expiration (slices 1 and 12), thereby improving the overall lung ventilation.

More importantly, these simulation results demonstrate that EIT is able to continuously track ventilation distribution in the lungs, and thus can be effectively used to adjust the level of PEEP that is sufficient to prevent alveolar collapse during lung deflation.

## V. Assessment of Lung Collapse Using EIT

**Fig. 13.**
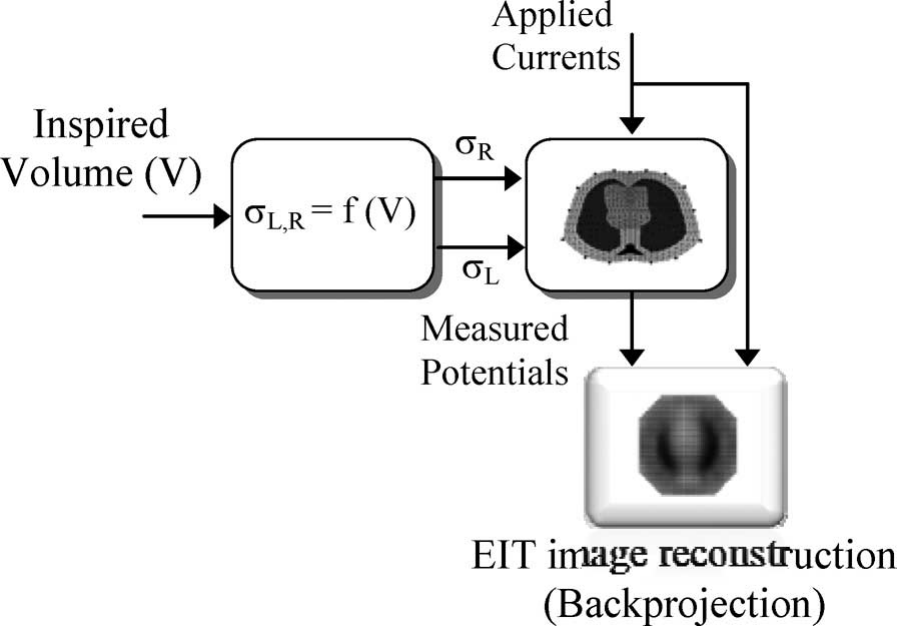
Simulation model based on the backprojection [5] image reconstruction algorithm.

This simulation study aims to illustrate the ability of EIT to detect collapsed regions of the lungs, which can be assimilated to the shunt fraction (alveoli that are perfused but not ventilated). The simplified model shown in Fig. 13 is used here to simulate inspiration cycles. The backprojection algorithm [5] is used for image reconstruction.

To simulate different degrees of lung collapse in ARDS, the bottommost (dorsal) units of the finite-element cross section of the lung model were grouped into three layers and assumed to be a mixture of well-conducting body fluids without any breathing activity.

**Fig. 14.**
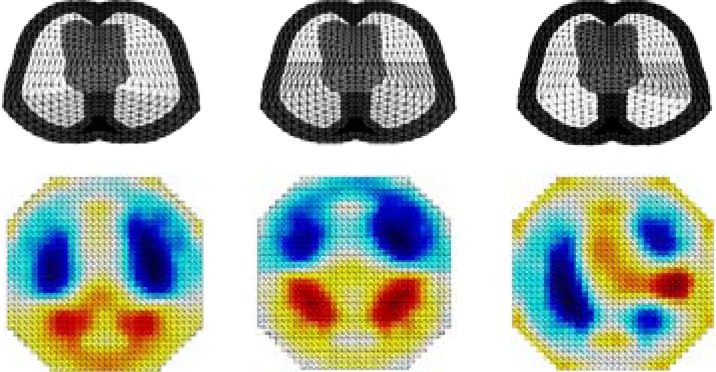
(Top panel) Simulated right and left lung conductivities changes and (bottom panel) collapsed lung regions with the reconstructed images.

Fig. 14 (top panel) shows the finite element mesh with three different collapsed region (in dark). Fig. 14 (bottom panel) depicts the lung model collapse and the reconstructed images at full inspiration. Dark blue regions in the bottom panel correspond to inflated lung units, whereas the red parts show the collapsed lung units.

This simulation model illustrates the behavior of EIT when detecting collapse or fluid shifts in damaged lungs and its potential to guide the titration of applied pressures during ventilator therapy. In particular, EIT may allow clinicians to achieve the best compromise when adjusting airway pressures to reduce overinflation of nondependent lung units and reinflate collapsed airways.

Clinical data are frequently affected by noise and will often need clinical interpretation to understand the cause of the abnormality that is seen, and a decision made should be as to how this should best be treated. There is also often a clinical debate as to the value of certain monitoring techniques in a practical situation, such as the value of pressure volume loops and how these can be interpreted. Inflexion points on pressure volume loops are an example of this. These occur when the pressure volume relationship changes, indicating either improved compliance because airways are held open with PEEP. Alternatively, a fall in the change in volume per unit pressure increase seen at higher pressures suggests worsening compliance as the lung becomes overinflated. However, these inflexion points may be very indistinct or may not be obvious at all. Some of these issues are indeed important when considering the potential of EIT because measures of pressure and volume provide information about the whole respiratory system; hence, information on regional changes may be lost in the data originating from both lungs. EIT has an advantage in that regional change can be analyzed and changes over time are easier and potentially safer to monitor than formal pressure volume loop assessment. Even when pressure volume loops are used in the development of EIT, and in order to help establish either normal values of resistivity at different levels of lung inflation, the ability to examine regional change adds another dimension to our ability to examine the lung.

In practice, imaging with EIT during either an expiratory pause or an inspiratory pause may allow more information on lung recruitment to be obtained, and it would be anticipated that one key use of EIT would be to allow visualization during the process of formal lung recruitment to see whether additional pressures actually can open up closed airways and whether additional PEEP can keep these airways open. This is potentially valuable as current methods such as compliance assessment or changes in $\hbox{PaO}_{2}$ reflect on the whole lung and may be affected by changes in cardiac output as intrathoracic pressures increase during the recruitment process. Continuous measurement during normal ventilation will provide information on regional atelectasis or overinflation and dynamic changes such as regional air trapping. Indeed, it could be argued that these are the pressures that the lungs are continually exposed to and so are the most important guide to adjustment of ventilator pressures. In this context, absolute EIT is very important, so that comparisons can be made against normal values. It may be that in future studies, aEIT will demonstrate that above a certain level of overinflation, more severe lung volutrauma can be expected. The ability to look at regions of the lung in this context may be very important.

## VI. Prospective EIT-Based Decision Support System for Ventilated Critical Care Patients

**Fig. 15.**
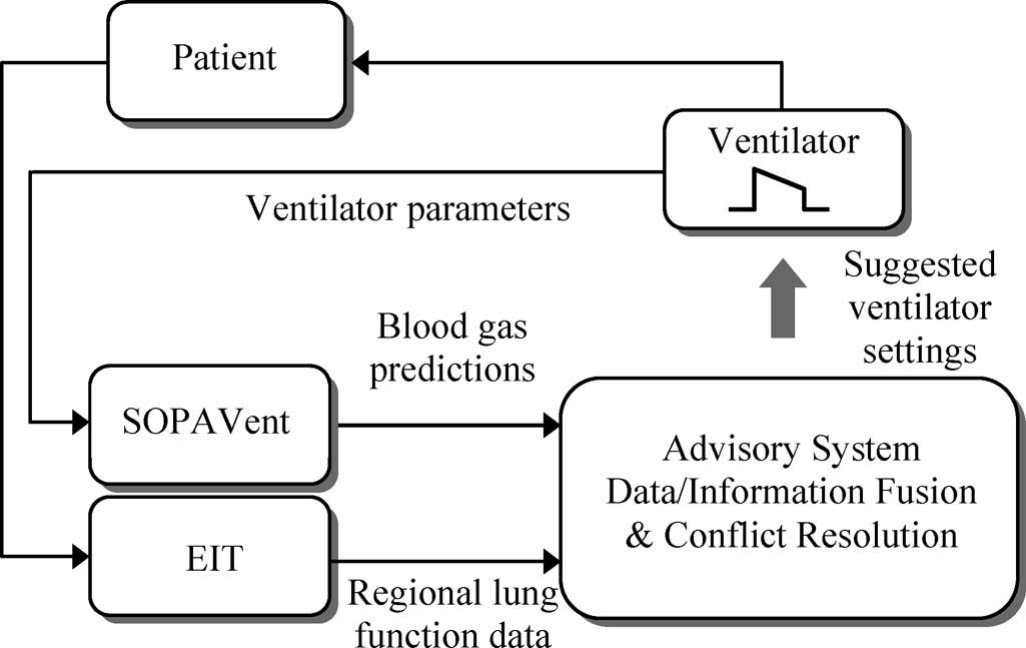
Structure of the proposed EIT-based advisory system for the management of critical care patients.

EIT is gradually gaining acceptance as a valuable tool for continuous and long-term monitoring of the regional lung function in critically ill patients. With this information available at the bedside along with other relevant patient's physiological parameters routinely monitored in ICUs, a computer advisory system can ultimately enhance the clinician's expertize with rapid and precise adjustments of ventilator settings, thus minimizing the known adverse effects of mechanical ventilation. The authors propose to use a previously developed data-driven physiological model (SOPAVent [27], [28]) for continuous and nonnvasive blood gas predictions, and the information generated from the Sheffield Mk3.5 absolute EIT [29] system to design the advisory system shown in Fig. 15. Key to the success of such a system will be data/information fusion block whose task will be to resolve any conflicts between the multisource nature of inputs of possible patient therapies prior to providing a unified decision.

## VII. Conclusion

EIT is an established monitoring technique with the potential to become a valuable bedside tool for the assessment of the pulmonary function. EIT is capable of tracking local changes in pulmonary air contents, and thus, can be used to continuously guide the appropriate setting of mechanical ventilation in critical care patients. A simulation model based on respiratory physiology has been developed to demonstrate the principles of EIT in monitoring ARDS-affected lung models under mechanical ventilation. The model is not intended to be a complete representation of the respiratory physiology of ventilated patients affected by ARDS. However, while relatively simple, it gives a good understanding of the processes of recruitment and derecruitment in ARDS-affected lungs, and illustrates how ventilator settings can be optimized with the aid of EIT monitoring techniques. The model was able to reproduce images of the ventilated lung under different ARDS conditions, which were consistent and presented a good agreement with the *PV* curve simulation results obtained for the same model of ARDS lungs. Future extension will include 3-D model representation for completeness of information. These models will be validated against clinical data recorded from ICU patients at the Northern General Hospital, Sheffield, U.K., using the Mk3.5 aEIT system.

The capacity of EIT imaging to pseudocontinuously assess the lungs' regional ventilation distribution at the bedside will undoubtedly offer new prospective opportunities and directions for the development of computerized decision support systems, which are expected to significantly improve the benefit-to-risk ratio of mechanical ventilation and delivery of care to critically ill patients. The authors also proposed a new advisory system architecture, integrating a noninvasive, continuously updated physiological model of blood gases with EIT data/information to provide advice for adjusting the ventilator parameters in future real-time clinical settings.

## Supplementary Material

Color versions of one or more of the figures in this paper are available online at http://ieeexplore.ieee.org.

## References

[1] Tremblay L. N. and Slutsky A. S., “Ventilator-induced lung injury: From the bench to the bedside,” in Intensive Care Med., vol. 32, pp. 24–33, 2006. 16231069 10.1007/s00134-005-2817-8

[2] Slutsky A. S., “Lung injury caused by mechanical ventilation,” in Chest, vol. 116, pp. 9S–15S, 1999. 10.1378/chest.116.suppl_1.9s-a 10424561

[3] Frank J. A. and Matthay M. A., “Science review: Mechanisms of ventilator-induced lung injury,” in Crit. Care, vol. 7, no. 3, pp. 233–241, 2006. 10.1186/cc1829 PMC270664 12793874

[4] Yilmaz M. and Gajic O., “Optimal ventilator settings in acute lung injury and acute respiratory distress syndrome,” in Eur. J. Anaesthesiol., vol. 25, no. 2, pp. 89–96, 2008. 18005469 10.1017/S0265021507003006

[5] Brown B. H., Barber D. C., and Seager A. D., “Applied potential tomography: Possible clinical applications,” in Clin. Phys. Physiol. Meas., vol. 6, pp. 109–121, 1985. 4017442 10.1088/0143-0815/6/2/002

[6] Brown B. H., “Electrical impedance tomography (EIT): A review,” in J. Med. Eng. Technol., vol. 27, no. 3, pp. 97–108, 2003. 12775455 10.1080/0309190021000059687

[7] Frerichs I., “Electrical impedance tomography (EIT) in applications related to lung and ventilation: A review of experimental and clinical activities,” in Physiol. Meas., vol. 21, pp. R1–R21, 2000. 10847187 10.1088/0967-3334/21/2/201

[8] Panoutsos G., Mahfouf M., Brown B. H., and Mills G. H., “Electrical impedance tomography (EIT) in pulmonary measurement: A review of applications and research,” in Proc. 5th IASTED Int. Conf., Biomed. Eng., Innsbruck, Austria, 2007, pp. 221–230.

[9] Victorino J. A., Borges J. B., Okamoto V. N., Matos G. F. J., Tucci M. R., Caramez M. P. R., Tanaka H., Sipmann F. S., Santos D. C. B., Barbas C. V. S., Carvalho C. R. R., and Amato M. B. P., “Imbalances in regional lung ventilation: A validation study on electrical impedance tomography,” in Amer. J. Respir. Crit. Care Med., vol. 169, pp. 791–800, 2004. 14693669 10.1164/rccm.200301-133OC

[10] Hinz J., Moerer O., Newmann P., Dudykevych T., Hellige G., and Quintel M., “Effect of positive end-expiratory-pressure on regional ventilation in patients with acute lung injury evaluated by electrical impedance tomography,” in Eur. J. Anaesthesiol., vol. 22, pp. 817–825, 2005. 16225714 10.1017/S0265021505001377

[11] Putensen C., Wrigge H., and Zinserling J., “Electrical impedance tomography guided ventilation therapy,” in Curr. Opin. Crit. Care, vol. 13, no. 3, pp. 344–350, 2007. 17468569 10.1097/MCC.0b013e328136c1e2

[12] Adler A. and Lionheart W. R. B., “Uses and abuses of EIDORS: An extensible software base for EIT,” in Physiol. Meas., vol. 27, pp. S25–S42, 2006, [Online]. Available: http://eidors3d.sourceforge.net/16636416 10.1088/0967-3334/27/5/S03

[13] Soni N. K., Paulsen K. D., Dehghani H., and Hartov A., “A 3-D reconstruction algorithm for EIT planner electrode arrays,” in IEEE Trans. Med. Imag., vol. 25, no. 1, pp. 55–61, Jan. 2006. 10.1109/tmi.2005.861001 16398414

[14] Holder D. S. Electrical Impedance Tomography: Methods, History and Applications (Series in Medical Physics and Biomedical Engineering), New York: Taylor & Francis, 2004.

[15] Bayford R. H., “Electrical impedance tomography,” in Annu. Rev. Biomed. Eng., vol. 8, pp. 63–91, 2006. 16834552 10.1146/annurev.bioeng.8.061505.095716

[16] Brandstatter B., Scharfetter H., and Magele C., “Multi frequency electrical impedance tomography,” in Comput. Math. Electr. Electron. Eng., vol. 20, no. 3, pp. 828–847, 2001.

[17] Hickling K. G., “The pressure-volume curve is greatly modified by recruitment: A mathematical model of ARDS lungs,” in Amer. J. Respir. Crit. Care Med., vol. 158, pp. 194–202, 1998. 9655729 10.1164/ajrccm.158.1.9708049

[18] Hickling K. G., “Best compliance during a decremental, but not incremented, positive end-expiratory pressure trial is related to open-lung positive en-expiratory pressure: A mathematical model of acute respiratory distress syndrome lungs,” in Amer. J. Respir. Crit. Care Med., vol. 163, pp. 69–77, 2001. 11208628 10.1164/ajrccm.163.1.9905084

[19] Salazar E. and Knowles J. H., “An analysis of the pressure-volume characteristics of the lungs,” in J. Appl. Physiol., vol. 19, pp. 97–104, 1964. 14104296 10.1152/jappl.1964.19.1.97

[20] Sundaresan A., Yuta T., Hann C. E., Chase J. G., and Shaw G. M., “A minimal model of lung mechanics and model-based markers for optimizing ventilator treatment in ARDS patients,” in Comput. Methods Programs Biomed., vol. 95, no. 2, pp. 166–180, 2009. 19327863 10.1016/j.cmpb.2009.02.008

[21] Crotti S., Mascheroni D., Caironi P., Pelosi P., Ronsoni G., Mondino M., Marini J. J., and Gattinoni L., “Recruitment and derecruitment during acute respiratory distress syndrome,” in Amer. J. Respir. Crit. Care Med., vol. 164, pp. 131–140, 2001. 11435251 10.1164/ajrccm.164.1.2007011

[22] Nopp P., Harris N. D., Zhao T. X., and Brown B. H., “Model for the dielectric properties of human lung tissue against frequency and air contain,” in Med. Biol. Eng. Comput., vol. 35, pp. 695–702, 1997. 9538548 10.1007/BF02510980

[23] Boyd E., Altman P. L., Dittmer D. S., Eds. Growth, including reproduction and morphological development in Biological Handbooks, Washington, DC: Federation of American Society for Experimental Biology, 1962, pp. 343–347.

[24] de la Grandmaison G. L., Clairand I., and Durigon M., “Organ weights in 684 adult autopsies: New tables for a Caucasoid population,” in Forensic Sci. Int., vol. 119, pp. 149–154, 2001. 11376980 10.1016/s0379-0738(00)00401-1

[25] Schiller H. J., Steinberg J., Halter J., McCann U., DaSilva M., Gatto L. A., Carney D., and Nieman G., “Alveolar inflation during generation of a quasi-static pressure/volume curve in the acutely injured lung,” in Crit. Care Med., vol. 31, no. 4, pp. 1126–1133, 2003. 12682483 10.1097/01.CCM.0000059997.90832.29

[26] Markhorst D. G., van Genderingen H. R., and van Vught A. J., “Static pressure-volume curve characteristics are moderate estimators of optimal airway pressures in a mathematical model of (primary/pulmonary) acute respiratory distress syndrome,” in Intensive Care Med., vol. 30, pp. 2086–2093, 2004. 15375648 10.1007/s00134-004-2446-7

[27] Wang A., Mahfouf M., and Mills G. H., “A continuously updated hybrid blood gas model for ventilated patients,” in Proc. 6th IFAC Symp. Model. Control Biomed. Syst., Reims, France, 2006, pp. 489–495.

[28] Kwock H. F., Linkens D. A., Mahfouf M., and Mills G. H., “SIVA: A hybrid knowledge-and-model-based advisory system for intensive care ventilators,” in IEEE Trans. Inf. Technol. Biomed., vol. 8, no. 2, pp. 161–172, Jun. 2004. 15217261 10.1109/titb.2004.826717

[29] Wilson A. J., Milnes P., Waterworth A. R., Smallwood R. H., and Brown B. H., “Mk3.5: A modular, multifrequency successor to the Mk3a EIS/EIT system,” in Physiol. Meas., vol. 22, pp. 49–54, 2001. 11236889 10.1088/0967-3334/22/1/307

